# Digital competence in health and nursing education: a cross-national comparison between samples from Germany and India using the DigKomp 2.2 questionnaire

**DOI:** 10.3389/frhs.2026.1801330

**Published:** 2026-04-17

**Authors:** Tom Schaal, Tim Tischendorf, Harshad Hend

**Affiliations:** 1 Faculty of Health and Healthcare Sciences, University of Applied Sciences Zwickau, Zwickau, Germany; 2 Harsh Institute of Nursing, Sutala Bk/Khamgaon/Buldana, Maharashtra, India

**Keywords:** cross-national comparison, DigComp 2.2, digital competence, digital health, health tech, nursing education

## Abstract

**Introduction:**

The digital transformation in healthcare requires future professionals to have solid digital skills. Despite the global relevance of digital skills, little is known about how these skills differ in countries with different healthcare and education systems. This study compares the self-assessed digital skills of two institutional samples of healthcare and nursing students in Germany and India using the DigKomp 2.2 questionnaire and examines the correlation between perceived competence and the strategies reported for searching for information.

**Methods:**

An online cross-sectional survey was conducted in April and May 2025 among 49 students in Germany and 256 students in India. Digital competences were assessed using the DigKomp 2.2 questionnaire, which covers all five dimensions of the DigComp framework. An open-ended question was used to record the students’ strategies for searching for reliable information on the internet. The quantitative data were analyzed using the Kolmogorov–Smirnov test, the Levene test, and the t-test for two independent samples.

**Results:**

Both cohorts showed acceptable to high internal consistency across all DigKomp 2.2 dimensions. Students in the German cohort reported higher competencies in data processing and evaluation and communication/collaboration, while students from the Indian cohort scored higher in digital content creation, security and problem solving. The exploratory bivariate analysis between the Indian and German samples revealed significant differences in 7 out of 15 items, specifically within the dimensions of digital content creation (3/3 items), Security (2/3 items), data processing & evaluation (1/3 items), and problem solving (1/4 items), whereas no significant differences were found in communication/cooperation (0/2 items). Qualitative responses revealed structured and academically oriented search strategies among German cohort. Indian responses showed greater heterogeneity.

**Discussion:**

Digital competence development varies substantially between the two institutional samples, reflecting differences in educational structures, access to digital technologies and national digitalization trajectories. While German students demonstrate strong academically grounded competencies, Indian students exhibit strengths in practical and applied digital competence. These findings underscore the need for context-sensitive digital education strategies that combine structured research competencies with practical digital literacy to prepare students for increasingly digitalized healthcare environments in both countries.

## 1 Introduction

Digitalization in healthcare and nursing has become an irreversible global trend that is shaping clinical practice, training, and professional roles in a wide range of healthcare systems. Digital technologies, ranging from information and communication systems to artificial intelligence (AI), generative AI (GenAI), robotic solutions, and digital monitoring, are increasingly being integrated into healthcare delivery and require corresponding skills from current and future healthcare professionals (1, 2). Digital competence is therefore not only a technical skill, but is closely linked to professional responsibility, ethical decision-making, and participation in digitally transformed healthcare ecosystems (3).

According to the European Union's definition, digital competence encompasses the confident and critical use of digital technologies for information, communication, and problem-solving in all areas of life (4). The DigComp 2.1 and later 2.2 frameworks have become widely used reference models for defining and specifically promoting such competencies in various areas, including health and education. In order to operationalize these competencies for empirical assessment, this study uses the DigKomp 2.2 questionnaire developed by Krempkow (5). The DigKomp 2.2 questionnaire (5), is based on the DigComp 2.1 descriptors (6) and translates the competence areas defined in the DigComp 2.1 framework into measurable survey items that enable self-assessment of digital competence across several dimensions.

Recent research shows that digital competence is not automatically acquired through frequent use of technology, especially among digital natives (7, 8). Rather, formal training is essential to transform existing lifestyle-related digital familiarity into workplace-relevant skills (2). This is particularly true in healthcare, where the safe, efficient, and ethically sound use of digital tools is required (7, 8). Global megatrends such as AI, digitalization, and globalization reinforce the need for the systematic development of digital competencies in healthcare education.

### 1.1 Digital health ecosystems in Germany and India

This paper presents an international comparison focusing on India. Its rapidly developing health and digital ecosystems represent a highly relevant point of comparison for Germany.

Germany has created a mature institutional framework for digital health, including electronic health records (EHR), electronic prescriptions, and a national telematics infrastructure (9). Despite this regulatory foundation, however, acceptance among citizens and providers remains relatively low (2). Less than one in five insured persons in Germany actively uses digital health services, with barriers such as interoperability issues, complex regulations, data protection concerns, and inconsistent implementation across regions (10, 11). This gap between infrastructural readiness and practical acceptance underscores the importance of strengthening the digital competencies of future healthcare professionals.

India is one of the world's most dynamic countries in the field of digital health, thanks to rapid digital expansion, large-scale public health programs, and strong government efforts toward digital transformation (12). A key development is the Ayushman Bharat Digital Mission (ABDM), which aims to create a nationwide digital health ecosystem that includes interoperable health records, unique health IDs, telemedicine platforms, and integrated registries. Digital health tools, such as teleconsultation services, AI-powered diagnostic applications, and mobile health platforms, are becoming increasingly prevalent in rural and urban areas thanks to the widespread use of smartphones (12). Nursing education in India has also undergone comprehensive reforms to integrate digital tools, simulation-based learning, and ICT-supported teaching strategies. However, challenges remain in terms of infrastructural differences between institutions, varying levels of digital literacy among teachers, and heterogeneous access to digital devices, especially in rural areas. These systemic differences shape the way nursing students develop, perceive, and apply digital competencies, making India an interesting point of comparison for Germany.

### 1.2 Relevance of cross-national comparison

The development of digital competencies is influenced not only by individual skills, but also by educational structures and national digital infrastructures. While Germany faces the challenge of putting progressive regulatory frameworks into practice (10), India is navigating rapid digital expansion in a highly diverse healthcare system with varying levels of access and institutional capacity. Examining the self-assessed digital competencies of health and nursing science students in these two contrasting systems can provide insights into how the digital transformation of health systems affects the development of individual competencies, insights into the influence of the digital transformation of health systems on the development of individual competencies, and findings for aligning educational strategies with national digitalization processes. A basis for designing competency-based curricula based on the EU's DigComp 2.2 framework (4). Furthermore, it can provide a deeper understanding of students’ readiness for future digital health environments. The survey examines not only self-assessed skill levels, but also the correlation between perceived competence and the maturity of students’ information search strategies (5). This mixed approach, which combines quantitative self-assessments with qualitative insights from open-ended responses, enables a nuanced examination of digital competence in different contexts.

### 1.3 Research objectives

Building on the EU's DigComp framework and the DigKomp 2.2 instrument, the present study explores digital competence among nursing and healthcare students in two institutional cohorts from Germany and India. The study addresses the following research questions: To what extent are there differences in the self-assessment of digital competence between the two groups of students with regard to the dimensions of digital competence according to the EU's DigComp framework? To what extent do self-assessments of digital competence correspond with the reported complexity of strategies used to search for reliable online information?

## 2 Methods

### 2.1 Questionnaire

Digital skills were assessed using the DigKomp 2.2 questionnaire (5), which represents a theoretically and empirically sound operationalization of the European DigComp 2.1 framework (6). The questionnaire consists of 15 items covering all five dimensions of digital competence – data processing and evaluation, communication and collaboration, digital content creation, security, and problem solving – and is rated on a five-point Likert scale from 1 (“not at all”) to 5 (“to a very great extent”) (5). Internal consistency was examined for each dimension at both the country level and for the overall sample using the Cronbach's alpha value.

In addition to the quantitative questions, the survey included an open-ended knowledge question. In this question, participants were asked to describe the strategies they typically use to find reliable online information. This allowed us to examine possible discrepancies between self-assessed competence and the complexity of the search behavior reported. As part of the coding process, two researchers independently reviewed all responses and developed inductively derived categories, which they refined through discussion to create a common codebook. In addition to the quantitative questions, the survey included an open-ended knowledge question. In this question, participants were asked to describe the strategies they typically use to find reliable online information. This allowed us to examine possible discrepancies between self-assessed competence and the complexity of the search behavior reported. As part of the coding process, two researchers independently reviewed all responses and developed inductively derived categories, which they refined through discussion to create a common codebook. This codebook contained category definitions, inclusion and exclusion criteria, and anchor quotes, and was then used for manual coding of the entire data set in Excel. Responses that did not contain interpretable qualitative content (e.g., Likert-type phrases such as “to a very high extent”) were considered invalid for qualitative analysis and were excluded from the coding procedure. In total, 20 responses (37,74%) in the Indian sample were excluded for this reason. The questionnaire for the German cohort was provided in the original version of DigKomp 2.2 according to Krempkow (5). For the Indian cohort, the questionnaire was translated into English based on the conceptual structure of the EU DigComp 2.2 framework. The translation aimed to preserve the semantic meaning of the original items and was reviewed by researchers familiar with the DigComp 2.2 framework and digital competence research. Minor adjustments were made to ensure linguistic clarity and contextual appropriateness for the Indian educational context. The questionnaire focused primarily on digital competence dimensions derived from the DigComp 2.2/DigKomp framework. Therefore, additional demographic and contextual variables (e.g., ICT experience or access to digital infrastructure) were not included in the survey instrument.

In addition to the core DigKomp 2.2 items used for the cross-cohort comparison, several context-specific questions were included in the Indian survey to capture aspects of digital learning conditions relevant to the local educational context. These additional items were analyzed descriptively and were not included in the main cross-cohort comparison.

### 2.2 Sampling

Sampling and data collection were carried out according to the predefined protocol. Local coordinators at the participating institutions distributed the link to the survey via the institutions’ mailing lists. The online questionnaire was hosted on SoSci Survey. Only fully completed questionnaires reaching the final page of the survey were included in the data analysis, ensuring that the dataset did not contain missing values for the closed-ended items. The open-ended question on information search strategies was optional, which explains the lower number of valid responses for this item. The German cohort consisted of 49 students from the Faculty of Health and Healthcare Sciences at the University of Applied Sciences Zwickau, while the Indian cohort consisted of 256 students enrolled in nursing or health studies at the Harsh Institute of Nursing. Participation was voluntary and and no personally identifiable information was collected. All respondents had to give their consent on the landing page before they could access the questionnaire. Those who did not consent were redirected to the end of the survey. The study was conducted in accordance with institutional research ethics guidelines. The Ethics Committee of the University of Applied Sciences Zwickau was consulted and confirmed that formal ethical approval was not required for this anonymous survey study. A detailed privacy policy was provided directly on the survey landing page, and all procedures complied with applicable data protection regulations. The survey period was identical for both cohorts, running from April to May 2025, with a single reminder email sent in the middle of the data collection phase.

### 2.3 Statistical analysis

Statistical analyses were conducted using IBM SPSS Statistics Version 30 for Mac. First, the Kolmogorov–Smirnov test was used to check whether the recorded variables were normally distributed. Next, the Levene test was used to test for variance homogeneity, which revealed no variance homogeneity. Against this background, a Welch test for two independent samples was performed. The bivariate statistical analysis was performed exploratively at item level between the two countries India and Germany; multiple testing did not take place—an alpha correction was not necessary. Internal consistency was assessed by calculating Cronbach's alpha for each of the five DigComp 2.2 dimensions at both the country level and for the overall sample (13).

## 3 Results

The survey was conducted among a total of 305 students. The sample consisted of 49 German students (*n* = 8 male; *n* = 40 female; *n* = 0 diverse; *n* = 1 no information) and 256 Indian students (*n* = 53 male; *n* = 187 female; *n* = 3 diverse; *n* = 13 no information). In addition, the course of study was surveyed for the Indian participants. 40.6% were enrolled in General Nursing Midwifery (GNM) (*n* = 104), 37.1% in Bachelor of Science Nursing (BSN) (*n* = 95), 14.8% were enrolled in Auxiliary Nursing Midwifery (ANM) (*n* = 38), and 1.6% (*n* = 4) were enrolled in other degree programs. 5.9% (*n* = 15) did not provide an answer. In addition, three questions were asked specifically to Indian students, addressing the use of digital applications in learning situations. These additional items were collected only in the Indian cohort and are therefore reported descriptively. They were not included in the main comparative analysis between the two institutional samples. The items were answered on a five-point Likert scale (1 = not at all, 5 = to a very large extent). In response to the question “To what extent can you identify the various file formats and their applications to use?”, 169 students gave an assessment, resulting in a mean value of 3.67 (SD ± 1.56). The question “To what extent can you use and connect audio video devices?” was answered by 166 participants and showed a mean value of 3.60 (SD ± 1.60). For the third question, “To what extent can you comfortably learn from the e-learning classes through applications such as GMeet, Zoom, etc.?” 164 students responded, with a mean value of 3.80 (SD ± 1.52). Cronbach's alpha coefficients were calculated for each of the five dimensions both at the country level and for the overall sample (Table 1).

**Table 1:** Internal consistency (cronbach's *α*) of the five DigKomp 2.2 dimensions across countries and for the total sample.

Dimension	Country (Cronbach's alpha coefficient)		Total
	Germany(mean value)	India(mean value)	
Data processing & evaluation	0.88	0.82	0.81
Communication/Cooperation	0.59	0.86	0.86
Digital content creation	0.80	0.84	0.83
Security	0.80	0.83	0.82
Problem solving	0.78	0.83	0.82

Most dimensions showed acceptable internal consistency, with Cronbach's alpha values above 0.70 and, in several cases, even above 0.8, indicating acceptable to good internal consistency. Specifically, coefficients above 0.7 are considered acceptable to good, while values above 0.8 are considered good. The only exception was observed in dimension 2 “Communication/Cooperation” for Germany, where the Cronbach's alpha value did not reach the threshold of 0.7. This value indicates limited reliability for this dimension within the German subgroup and should therefore be interpreted with caution. It should be noted that only fully completed questionnaires were included in the analyses, as all questions had to be answered before the online survey could be submitted. This result suggests that the measurement instruments used are highly reliable in each national context, thus supporting the validity of cross-cultural comparisons. The comparable alpha values confirm that the scales function consistently and appropriately regardless of cultural differences, enabling a meaningful interpretation of the results. Consequently, the measurement instruments used in this study are both culturally equivalent and psychometrically robust, facilitating a valid comparison of the constructs and ensuring that observed differences in responses can be reliably attributed to content factors rather than methodological inconsistencies.

**Figure 1:**
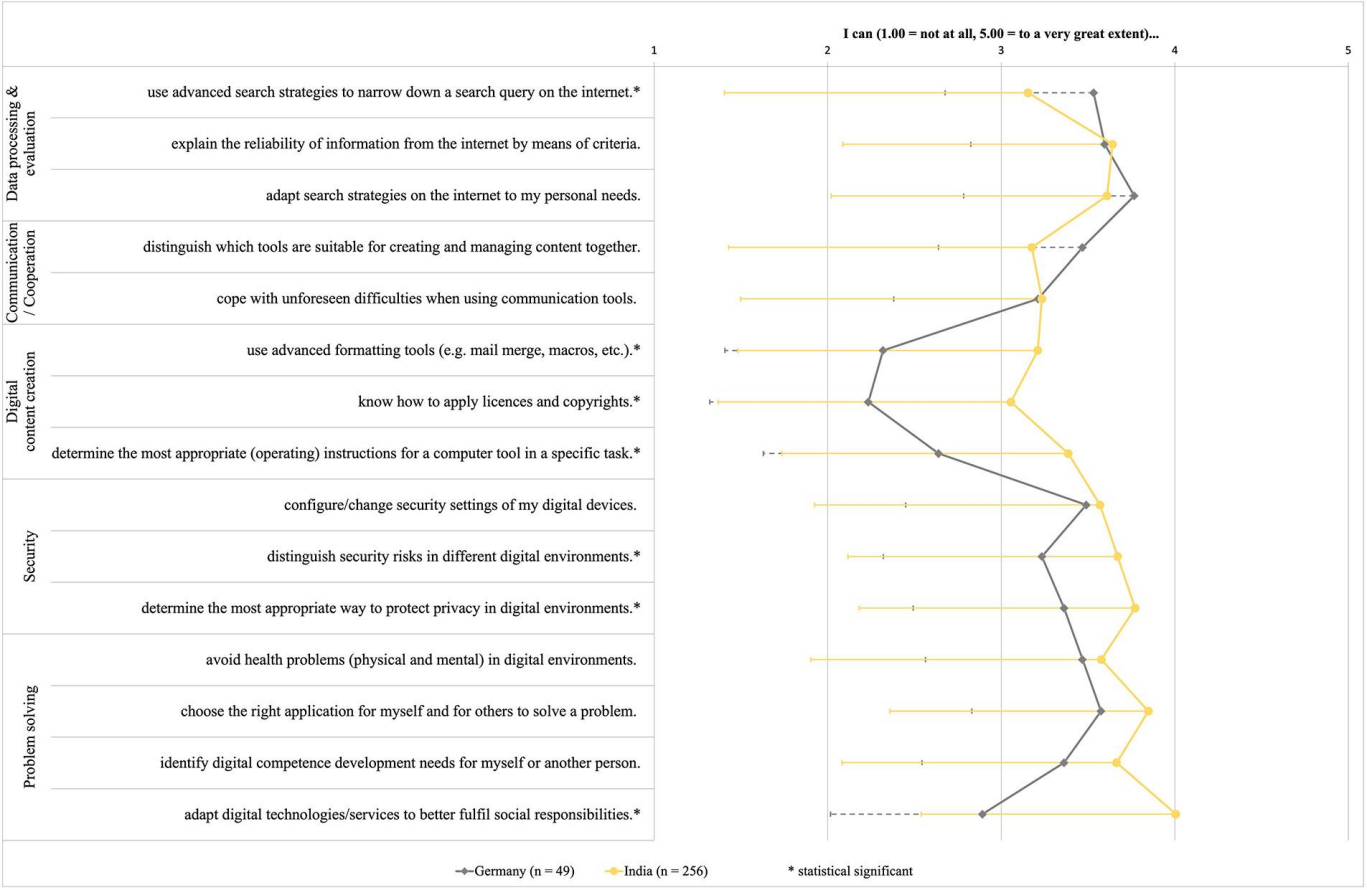
Mean (negative value of standard deviation) values of the digital competencies recorded (*difference statistically significant). 1.00 = not at all, 5.00 = to a very great extent.

The digital competence related variables were not normally distributed and exhibited heterogeneous variances. Overall, differences in mean values of digital competencies were significant for 7 out of 15 items (Figure 1). Exact *p*-values, confidence intervals, effect sizes and standard errors are provided in the e-supplement, along with the dataset for download (e-supplement 1-2).

Furthermore, respondents were asked to describe the strategies they use to find reliable information online in an open-ended knowledge question related to the first dimension (data processing/evaluation). Responses from Germany (*n* = 18) and India (*n* = 52) revealed shared patterns as well as country-specific nuances. The qualitative responses were grouped into several categories reflecting different levels of information search strategies. These included, for example, basic search strategies, such as relying primarily on general search engines, and more advanced strategies, such as consulting scientific databases or cross-checking multiple sources. For instance, a typical response categorized as a basic strategy was: “I usually search for information using Google.” In contrast, more advanced responses referred to strategies such as “checking several sources and using scientific articles or medical databases.” All responses were anonymized before analysis. Participants from Germany described highly systematic and academically rigorous strategies. The most frequently mentioned approach was conducting systematic literature reviews (*n* = 8), often guided by frameworks such as RefHunter or the PICO scheme. As one student put it: ’Systematic literature search (10 steps according to RefHunter)’. Boolean operators (AND, OR and NOT) were commonly used in databases such as PubMed and Google Scholar (*n* = 6), with responses such as ‘Keywords, key terms, Boolean operators (AND/OR/NOT)’. The snowball method of tracing citations from relevant articles was also employed (*n* = 4). Other recurring strategies included using scientific databases and official sources such as national statistics portals, as well as formulating search strings using keywords, synonyms and wildcards. Some participants mentioned using ChatGPT to generate keywords and reflected on the growing role of AI tools in replacing manual search processes.

In contrast, the 53 Indian responses showed significantly greater heterogeneity. The term “Google” or its variants (e.g., “Google.com,” “Google Chrome”) appeared in 7 responses, making it the most frequently mentioned explicit tool. The general term “Internet” appeared in 4 responses. Short, unspecific entries such as “learning” were common (*n* = 5), as were negations or minimal responses such as “no,” “not,” “not at all” (*n* = 5). A striking pattern was the repeated use of the Likert scale phrase “To a very high extent,” which appeared in 20 responses. This repetition strongly suggests that some students misunderstood the open-ended question and carried over their response behavior from the previous rating scale items. A proportion of responses in the Indian sample consisted of Likert-type expressions rather than descriptive statements and were therefore excluded from the qualitative analysis. Despite the prevalence of minimal or general responses, a smaller portion of the Indian cohort mentioned more advanced strategies. Detailed techniques, including Boolean operators, identifying key concepts, using synonyms, truncation, or phrase searching, appeared in 3 responses. In addition, explicit statements were made that referred generally to “advanced search strategies” (*n* = 5), suggesting that at least some awareness of more systematic search behaviors existed within the group.

In summary, it can be said that the German cohort consistently presented methodologically sound search strategies based on the use of academic databases and structured procedures. The Indian cohort, on the other hand, showed a broader spectrum – from basic, tool-oriented approaches to a small number of more advanced techniques. The high frequency of unspecific or scale-like responses suggests that many students may have limited experience with structured literature searches or may have had difficulty with the open format.

In addition, mean values were calculated and tabulated for each of the five dimensions of digital competence according to DigKomp 2.2 questionnaire for further comparison (Table 2).

**Table 2:** Overview of mean values per dimension of digital competencies.

Dimension	Country (mean value)	
	Germany	India
Data processing & evaluation	3.63	3.43
Communication/Cooperation	3.34	3.11
Digital content creation	2.40	3.10
Security	3.36	3.67
Problem solving	3.33	3.74

1 = not at all, 5 = to a very great extent.

## 4 Discussion

This research employed the DigKomp 2.2 questionnaire (5) to rigorously assess self-perceived digital competencies among healthcare and nursing students in Germany and India. Findings revealed both commonalities and pronounced disparities between the two institutional samples, which can be attributed to the distinct trajectories of digital transformation in their respective health and education sectors. Importantly, the five DigKomp 2.2 dimensions achieved acceptable to high internal consistency levels across both countries, confirming the instrument's reliability in diverse cultural contexts. A possible explanation for the lower Cronbach's alpha value for Germany in the Communication/Cooperation dimension may be that, in the Indian sample, responses tended to be more uniform, resulting in higher inter-item correlations and thus increasing Cronbach's alpha; additionally, the English translation of the items for India may have led to clearer and more consistent responses. The robustness of these psychometric properties, despite the lower value observed in one dimension, generally suggests that the DigComp 2.1/2.2 framework remains a valid basis for meaningful international comparisons.

The German students cohort exhibited higher self-assessed competencies in data processing, evaluation, communication, and collaboration. These outcomes likely derive from stable academic infrastructures, standardized digital learning protocols, and curricula prioritizing source criticism and systematic information management (3). Conversely, the Indian cohort showed enhanced capabilities in digital content creation, security, and problem solving, potentially reflecting the rapid digitalization of India's healthcare sector and extensive adoption of mobile technologies and e-learning platforms (12).

The qualitative responses provide additional context for interpreting the quantitative findings. In several cases, students who reported high levels of digital competence described relatively basic strategies for locating reliable information online, such as relying primarily on general search engines. This suggests that self-assessed competence may not always correspond to more advanced information-search strategies. At the same time, some responses demonstrated more structured approaches, such as cross-checking multiple sources or consulting scientific databases. These qualitative insights help contextualize the quantitative self-assessments and highlight potential discrepancies between perceived and applied digital competence. German students frequently described systematic, academically grounded search strategies, including structured literature reviews, Boolean logic, and use of institutional databases, emphasizing strong evidence-based research integration. Indian responses, however, ranged from general use of search engines to demonstrations of advanced search skills by a minority, with a significant portion offering brief or survey-like answers, possibly indicating challenges in articulating strategies or disparities in search expertise due to linguistic, educational, or structural factors. Consequently, the qualitative data provide exploratory insights rather than fully comparable evidence between the two institutional samples.

Further data from descriptive questions unique to the Indian cohort highlighted intra-group differences. While average performance in recognizing file formats, operating audio-video devices, and engaging in e-learning was moderate to high, large standard deviations suggested variable familiarity and confidence levels - likely a result of infrastructural and technological disparities within India's educational landscape (12). However, given the exploratory design and the comparison of two institutional samples, these interpretations should be understood as tentative.

Taken together, these results emphasize the necessity of context-sensitive strategies for developing digital competencies in nursing and health education (2). The distinction between academically oriented and practice-based digital skills supports the integration of both structured research methods and applied digital literacy within curricula to better prepare students for increasingly digital healthcare environments in both countries (14). Interdisciplinary collaboration, engaging healthcare professionals, IT experts, and educational researchers, offers promising potential for designing robust frameworks that address the multifaceted nature of digital competence (4). Although the findings are exploratory and based on two institutional samples, they provide preliminary insights that may inform future educational initiatives. Future research should evaluate the impact of such collaborations on curriculum design, student engagement, and professional outcomes.

Additionally, faculty development emerges as a crucial factor in promoting digital competence. As key agents in technology integration, educators require targeted training to close gaps in pedagogical and digital knowledge (15). The effectiveness of varied faculty development models across different cultural contexts warrants investigation to ensure both instructors and students are well-equipped for ongoing digital transformation in healthcare education.

Lastly, future studies should consider the influence of emerging technologies, such as artificial intelligence and virtual reality - on the evolution of digital competencies. Integrating these innovations into educational programs holds promise for enhancing technical proficiency and fostering adaptability and critical thinking. Comparative cross-national research into the implementation and evaluation of such tools may yield valuable guidance for best practices globally.

## 5 Limitations

Several methodological limitations should be taken into account when interpreting the findings of this study. Firstly, the reliance on voluntary participation via institutional mailing lists may have introduced self-selection bias, potentially favoring students with a stronger interest in digital topics. This bias could influence the mean levels of reported digital competencies. Secondly, while the online questionnaire required completion of all items prior to submission, the use of a non-personalized survey link means that duplicate participation cannot be entirely ruled out, although no patterns indicative of such occurrences were detected. Thirdly, the application of self-assessment measures entails the risk of subjective bias, as perceived competence may not accurately reflect actual ability. Discrepancies due to either overestimation or underestimation of digital skills are therefore possible. Although a qualitative free-text item was included to assess applied competence, the responses differed markedly in depth and interpretability across cohorts. In the Indian group, the frequent occurrence of short or scale-like answers suggests some participants may have misunderstood the open-ended format, potentially limiting the comparability and richness of the qualitative data. The lower internal consistency observed for the communication and collaboration dimension in the German sample may be related to the small subgroup size and the limited number of items representing this construct. Therefore, differences between the two institutional samples in this dimension should be interpreted cautiously.

A further limitation concerns the cross-cultural comparability of the instrument. Although the DigKomp 2.2 instrument (5) yielded strong internal consistency in both countries, subtle cultural and linguistic factors could affect the interpretation of individual items. For the Indian cohort, the translation of the questionnaire into English based on the DigComp 2.2 framework (4) was conducted with care; nonetheless, contextual nuances might have influenced response behavior. Although the questionnaire was translated carefully and aligned with the DigComp framework, formal tests of measurement invariance between the German and Indian samples were not conducted. Therefore, differences between the two institutional samples should be interpreted cautiously and understood as exploratory indications rather than definitive evidence of cross-cultural differences in digital competence. Additionally, differences in institutional digital infrastructure, exposure to digital technologies, and pedagogical approaches between Germany and India may impact self-assessment patterns in ways that the questionnaire cannot fully capture.

Another limitation concerns the imbalance in sample sizes between the two cohorts (Germany *n* = 49, India *n* = 256), which may affect statistical comparability and the stability of some estimates. In addition, the survey did not collect detailed information on potential confounding variables such as age, semester level, prior ICT experience, or access to digital resources. These factors may influence digital competence and self-assessment patterns and should be considered in future research.

Finally, the cross-sectional nature of the study prohibits the inference of causality. The results provide insight into digital competence at a specific point in time but do not account for long-term development or the effects of curriculum changes and infrastructural investments. The findings are based on two institutional samples and therefore cannot be generalized to national contexts. Differences observed in this study should be interpreted as exploratory comparisons between two educational cohorts. Future research should include multicenter sampling across institutions, longitudinal designs, and objective assessments of digital competence (e.g., performance-based tasks) in order to strengthen the validity and generalizability of findings.

## Data Availability

The raw data supporting the conclusions of this article will be made available by the authors, without undue reservation.
