# Supplementary material for: Digital competence in health and nursing education: a cross-national comparison between samples from Germany and India using the DigKomp 2.2 questionnaire
Source: Front Health Serv. 2026 Apr 17;6:1801330. doi: 10.3389/frhs.2026.1801330 (PMC13132836; doi:10.3389/frhs.2026.1801330)
Supplement: Supplementary file 3 [file Datasheet2.pdf]

Page 01

Questionnaire Error

There is no text with the ID **Start** in this survey project.

Dear participants,

thank you for your interest in our survey. Following is an assessment of your digital media and technology skills according to the EU reference framework for digital competence (DigComp2.1).

We would be grateful if you could take part in the survey. Your answers will be completely anonymous. It is not possible to draw any conclusions about you as an individual.

Page 02

1. What is your gender identity? D001

- ○ male
- ○ female
- ○ other ______

- ○ no answer

2. What degree program are you currently enrolled in? D004

- ○ Auxiliary Nursing Midwifery (ANM)
- ○ General Nursing Midwifery (GNM)
- ○ Bachelor of Science Nursing (B.Sc.)
- ○ other ______

3. To what extent are the following skills currently developed in you?

D101

I can...

	not at all				to a very large extent
use advanced search strategies to narrow down a search query on the internet.	○	○	○	○	○
explain the reliability of information from the internet by means of criteria.	○	○	○	○	○
adapt search strategies on the Internet to my personal needs.	○	○	○	○	○

4. What search strategies do you use for this?

D102

5. To what extent are the following skills currently developed in you?

D201

I can...

	not at all				to a very large extent
distinguish which tools are suitable for creating and managing content together.	○	○	○	○	○
cope with unforeseen difficulties when using communication tools.	○	○	○	○	○

6. To what extent are the following skills currently developed in you?

D301

I can...

	not at all				to a very large extent
use advanced formatting tools (e.g. mail merge, macros, etc.).	○	○	○	○	○
know how to apply licences and copyrights.	○	○	○	○	○
determine the most appropriate (operating) instructions for a computer tool in a specific task.	○	○	○	○	○

D401

7. To what extent are the following skills currently developed in you?

I can...

	not at all				to a very large extent
configure/change security settings of my digital devices.	○	○	○	○	○
distinguish security risks in different digital environments.	○	○	○	○	○
determine the most appropriate way to protect privacy in digital environments.	○	○	○	○	○

D501

8. To what extent are the following skills currently developed in you?

I can...

	not at all				to a very large extent
avoid health problems (physical and mental) in digital environments.	○	○	○	○	○
choose the right application for myself and for others to solve a problem.	○	○	○	○	○
identify digital competence development needs for myself or another person.	○	○	○	○	○
adapt digital technologies/services to better fulfil social responsibilities.	○	○	○	○	○

ZU01

9. Please answer the following questions.

	not at all				to a very large extent
To what extent can you identify the various file formats and their applications to use?	○	○	○	○	○
To what extent can you use and connect audio video devices?	○	○	○	○	○
To what extent can you comfortably learn from the E-learning classes through applications such as Gmeet, Zoom etc.?	○	○	○	○	○

Thank you for completing this questionnaire!

We would like to thank you very much for helping us.

Your answers were transmitted, you may close the browser window or tab now.

Tim Tischendorf, Faculty of health and healthcare sciences, West Saxon University of Applied Sciences Zwickau, Germany – 2026
